# Development and Integration of Community Advisory Boards to Inform Research on the Impact of Structural Racism on Racial/Ethnic Disparities in End‐Stage Kidney Disease

**DOI:** 10.1111/hex.70406

**Published:** 2025-08-27

**Authors:** Andrea Corradi, Grecia B. V. Meléndez, Tanjala S. Purnell, Selena E. Ortiz, Catherine R. Butler, Jonathan Daw

**Affiliations:** ^1^ Department of Criminal Justice and Criminology Georgia Southern University Statesboro Georgia USA; ^2^ Department of Health Policy & Management Johns Hopkins Bloomberg School of Public Health Baltimore Maryland USA; ^3^ Departments of Epidemiology and Surgery Johns Hopkins Bloomberg School of Public Health and Johns Hopkins School of Medicine Baltimore Maryland USA; ^4^ Department of Health Policy the Pennsylvania State University University Park Pennsylvania USA; ^5^ Department of Medicine and the Kidney Research Institute, Division of Nephrology University of Washington Seattle Washington USA; ^6^ Nephrology Section, Hospital and Specialty Medicine, and Seattle‐Denver Health Services Research and Development Center of Innovation Veterans Affairs Puget Sound Health Care System Seattle Washington USA; ^7^ Department of Sociology & Criminology the Pennsylvania State University University Park Pennsylvania USA

**Keywords:** community, kidney disease, lived experiences, patient‐centered, qualitative

## Abstract

**Introduction:**

Community Advisory Boards (CABs) represent a critical methodological approach to center research on the experiences of patients and families and minimise power inequities between ‘researcher’ and ‘subject’. This approach can be especially important to inform work related to structural racism and racial and ethnic disparities in healthcare. Herein, we report the value and challenges of integrating CABs into ongoing research on structural racism and racial and ethnic disparities in care for adults with end‐stage kidney disease as well as strategies to maximise impact.

**Methodology:**

CABs were assembled to inform a large mixed‐methods research project focused on the impact of structural racism on end‐stage kidney disease. Qualitative data derived from CAB deliberation were analysed with constructivist grounded theory and used to contextualise research findings and evaluate intervention points.

**Results:**

Two CABs included 21 adults with kidney disease, caregivers and community members in Pittsburgh and Baltimore. CAB member reflections on research presentations were collected, collated and integrated into ongoing research at multiple stages of development. Further, the members were engaged in the iterative development of the CAB structure itself—including format of meetings from semiannual gatherings to more frequent ad hoc combined meetings—based on member feedback.

**Conclusions:**

Our experience identifies opportunities to facilitate a critical shift in the role of patients and community members as study participants to become collaborators in health research and practice.

**Patient or Public Contribution:**

CAB affiliates, who are members of the end‐stage kidney disease community, can provide feedback on analyses, suggest and drive lines of inquiry, and contribute to methodological changes including suggesting more frequent meetings. They are collaborators rather than study subjects.

## 1 Introduction

Community Advisory Boards (CABs) offer a valuable forum for collaboration between patients, community members, clinicians and researchers. This study approach provides a unique opportunity for collecting data about lived experiences, designing grass‐roots interventions and initiatives, as well as helping to close the divide between academia and practice [1, 2]. Community‐based research promotes ‘multiple ways of knowing’ [3] and CABs have been shown to be effective forms of academic‐community partnerships, especially in understanding and addressing health disparities [4, 5]. This community‐centered approach is especially important when research relates to social determinants of health and the role of structural racism in health disparities; since patient communities bring lived experiences, differing perspectives and insights that expand on the often‐siloed perspectives of academia.

Historically, health services research focused on kidney disease has largely centered around the evaluation of patient cohort data, national registries and medical claims data, traditionally following a top‐down approach in which investigators frame study questions, design and interpretation of findings on behalf of the community being studied. Although those affected by study findings—patients and their loved ones—may be included as research subjects, they often have little opportunity to shape the approach and direction of the work. Increasing recognition of substantial gaps in the priorities, values and perspectives between clinicians, researchers and the communities they serve underscores the limitations to standard research practice.

In recent years, many marginalised groups including indigenous peoples and individuals with disabilities have adopted the perspective of ‘nothing about us, without us’, emphasising the need to include the viewpoints of those whose voices and lived experiences have historically been ignored or silenced by traditional, hierarchical research design [6]. The foundational aim of CAB methodology is to reconstruct research practice to achieve such an outcome. People affected by phenomena being studied are invaluable, not only as subjects, but at all stages of research. Integrating community members into the research team both empowers the community being studied and grounds the research in lived experience.

We herein offer an example of the value, development and integration of our CAB approach focused on structural racism and disparities in kidney disease as a core component of an NIH‐funded large research study.^1^ Our study examines the role of structural racism in the experience and outcomes of people with end‐stage kidney disease (ESKD). To better understand this complex phenomenon, we triangulate multiple methodologies including secondary and multilevel quantitative analyses, qualitative focus groups and quantitative‐qualitative concurrent mixed‐methods analyses. Mixed methods studies, especially those adopting an advocacy and transformative focused lens, are underutilized but impactful [7]. Our CABs occupy a central role in helping to guide this complex research program. The group engages in the formation of research questions, and discussion of research findings and potential interventions. The overarching goal is to close the feedback loop, in which we employ findings from our CABs to inform analyses, and we thereafter present findings to the CABs for comment and contextualisation. To this end, we document the experiences of our CAB participants as practitioners, community leaders, patients and their support partners throughout the research process.

## 2 Background

### 2.1 Structural Racism and ESKD

For decades, there has been mounting evidence of stark and persistent racial disparities in access to care, experiences, and clinical outcomes for people with ESKD in the United States [8, 9, 10, 11, 12, 13]. These racial disparities have manifested in a range of complex and overlapping ways. For example, the racial composition of a community, specifically a higher percentage of Black residents, is linked to both mortality and hospitalisation in patients who receive maintenance dialysis even after controlling for relevant patient and health system characteristics [14]. Furthermore, Black patients were shown to be less likely to receive timely pre‐ESKD nephrology care compared with other racial groups, regardless of where they lived [15, 16]. Black patients in communities with lower than average incomes and in which the majority of residents are Black are less likely to appear on deceased donor transplant waitlists as compared with those living in majority White neighbourhoods [17]. Along with demographic‐level differences, racism at interpersonal‐, institutional‐, and public policy‐levels contributes to racial differences in outcomes along the spectrum of kidney disease [18].

Structural racism can be described as a subdomain of systemic racism, that is, “a multifaceted, interconnected and institutionalised system of relational subordination for people of colour and superordination for non‐Hispanic White people that is observable as manifest racial inequalities in life chances” [19, 20]. Structural racism contributes to disparities that are not the product of individual decisions but rather are ‘baked in’ to a network of mutually reinforcing and justifying institutional practices that influence one another dynamically over time. Structural racism has been connected to disparities in a variety of institutions in society such as the criminal justice system [21]. Repeated contact with these institutions has been shown to have compounding negative health outcomes [22]. Therefore, racialized power structures, and the legacies of disadvantage inherent in them, cannot be ignored when examining racial disparities in ESKD [23].

Disparities in health can be both a component of structural racism or be caused by dimensions thereof [24]. For example, legacies of unequal distribution of socioeconomic resources contribute to disparities in access to health insurance, which affect present‐day socioeconomic conditions [25], and employer‐based insurance coverage [26]. Redlining—the process that flagged communities with large Black populations as areas too hazardous to receive federal loans to make housing more affordable—resulted in continuing residential segregation, and lower transfer of intergenerational wealth [27]. These historical discriminatory investments in community access to homeownership, which are associated with present‐day racial‐ethnic residential segregation in many metropolitan areas, continue to contribute to health conditions today [28]. The goal of our research study is to examine how structural racism contributes to racial disparities in access to care and outcomes among people living with ESKD specifically.

### 2.2 CABs

The value of community‐academic partnerships for research is well established. For example, since 2004, the state of Wisconsin has been funding programs that must include a community health partner and an academic one [29]. Many community‐academic partnerships focus primarily on social determinants of health [3] as lived experiences can provide significant information and grounding in this area of public health specifically. An emphasis on ‘multiple ways of knowing’ is crucial when highlighting the role that patient‐centered research can play. Though community partnerships often face challenges of sustainability and time‐constraints, they can be incredible sources of collaboration and strategic planning [29].

CABs (also known as patient advisory boards in some contexts) have been used to study issues such as cardiovascular health disparities [4], the dissemination of organ donation information [30, 31], living donor kidney transplantation [31], spinal‐cord injuries and diabetes [5, 32]. Often CABs may be used to design interventions [30] rather than to guide ongoing health services research processes as they are here. When community‐academic partnerships take the role of CABs to inform and contextualise analyses beyond the intervention stage, a community‐based participatory framework is frequently used. To align with this framework, it is crucial to involve CABs equitably and to include them throughout all phases of research [4]. This framework has been found to enhance trust and quality of data, create culturally appropriate tools of measurement, and deepen connection to impacted communities to ultimately create responses that are adaptive to its needs [33]. Another framework often associated with CABs is consumer and community engagement, which also prioritises shared decision‐making and participation in research [34]. Regardless of the framework, best practices for CABs include defining roles and operating functions, having a clear recruitment strategy, and using multiple methods of data collection [32].

## 3 Methods

### 3.1 Data Collection

Our research team is inter‐ and multidisciplinary (i.e., sociology, criminology, medicine, public health), consists of collaborators from 12 universities, and employs a variety of national datasets and methodological approaches to carry out the project's core aims. We have been able to develop two CABs, representing two different medium‐sized metropolitan cities located in the Central Atlantic states with a documented history of structural racism—Baltimore, MD and Pittsburgh, PA [35, 36]. In the first stages of the project, CAB meetings were held semi‐annually with each group in the spring and fall seasons. A joint CAB that meets more frequently was in development before grant cancellation. CABs participants include adults with ESKD, living kidney donors, their caregivers and family, social workers, medical doctors, faith‐leaders and community organisers. CAB meetings were held virtually for a number of reasons including protecting the well‐being of immune‐comprised members, eliminating transportation barriers, facilitating the integration of researchers across the country, and adapting to the findings of previous research on CABs that rurality can be a significant barrier to participation [37]. Virtual meetings span approximately 2 h in length, and participants are provided $50.00 USD per meeting in appreciation for their time and effort.

**Figure 1:**
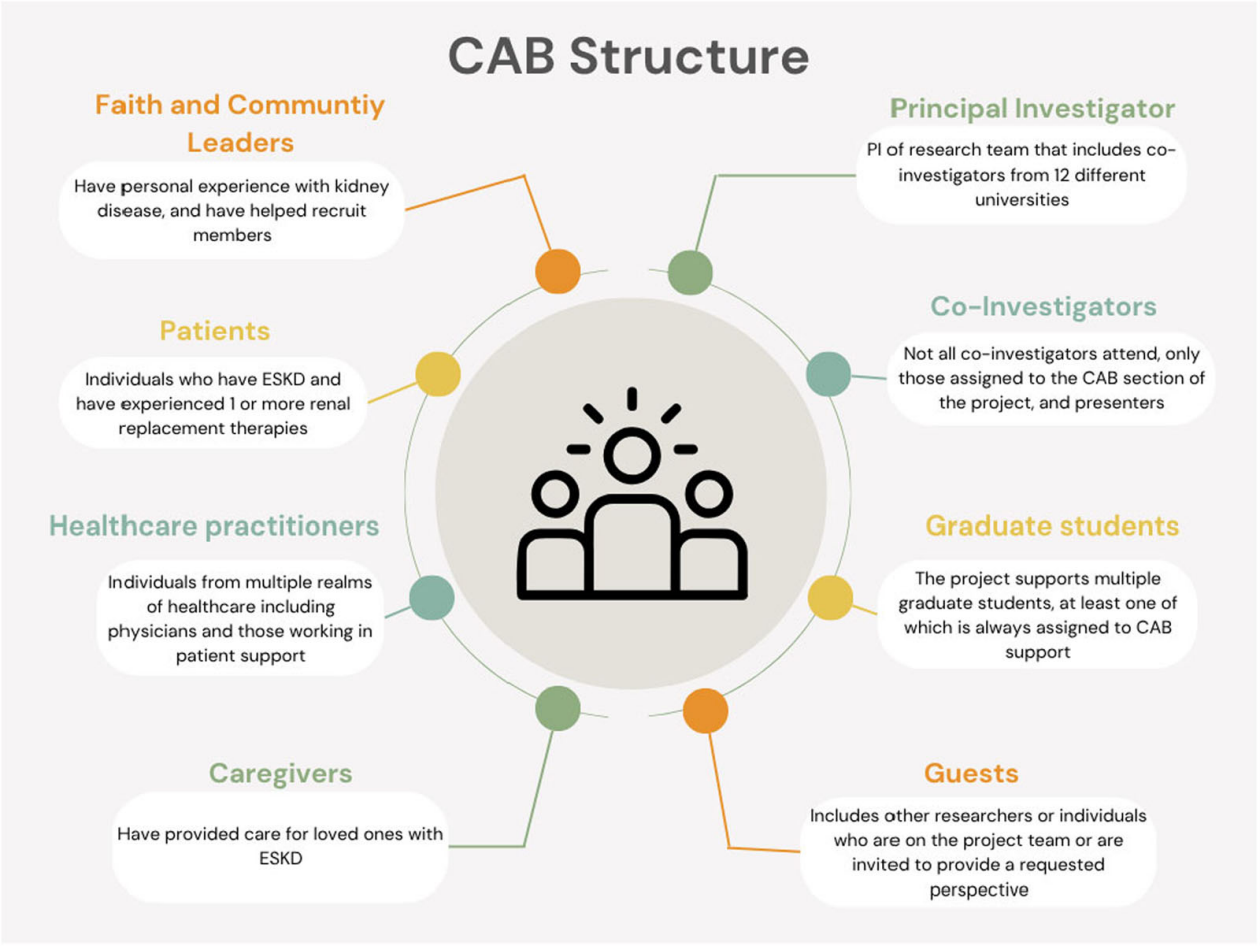
CAB structure.

We view and value the CABs as another member of the research team; their suggestions and ideas are heeded. It is crucial to our process that study findings are presented for feedback, not only for informational purposes. Therefore, all lines of inquiry are presented to the CABs before manuscript completion at a minimum but ideally are presented early in the research process to inform the research design from start to finish (Figure 1).

**Figure 2:**
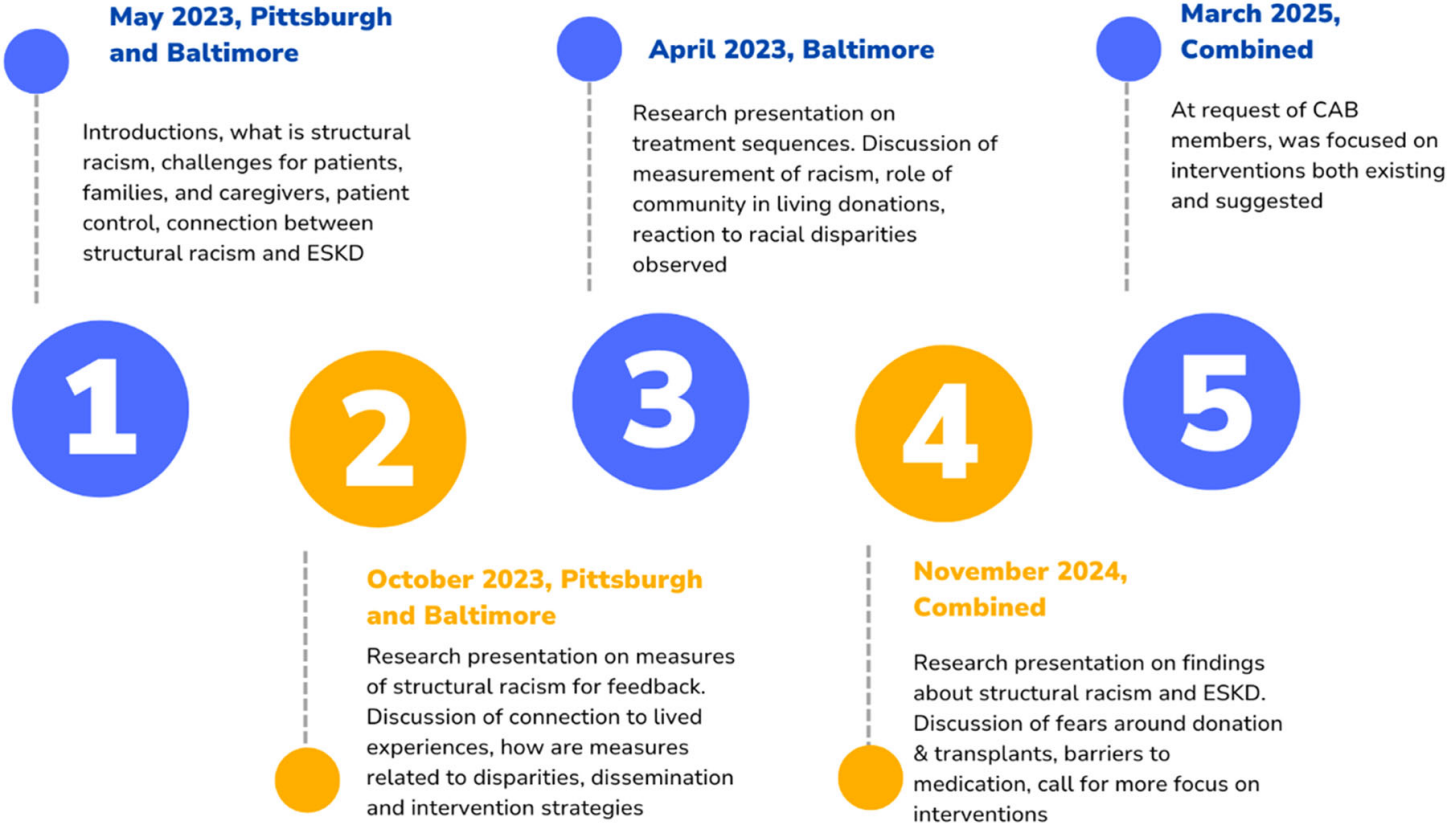
Timeline and topics of meetings.

The first CAB (CAB1) is primarily based in the Baltimore Metropolitan Area. The city of Baltimore, which has the 11th largest Black population in the United States [38], has a long history of successful partnerships between academic researchers and community advisors. The initial recruitment approach for members of CAB1 members included outreach to existing members of an ongoing community‐based participatory research group, ‘The Faith‐Based Alliance for Kidney Health and Transplant Equity’. The Faith‐Based Alliance for Kidney Health and Transplant Equity is directed by Dr. Tanjala Purnell and was initially founded in 2019 [31]. A total of 15 people accepted the invitation to join CAB1. The Institutional Review Board at Johns Hopkins School of Medicine approved the project activities as exempt from review (IRB00187277). To date, CAB1 has met virtually a total of five times (May 2023, October 2023, April 2024 and November 2024, March 2025) (Figure 2).

The second CAB (CAB2) is based in Pittsburgh, PA, and is a relatively new group that was organised in 2022 for the purposes of this study. A total of six people accepted the invitation to join CAB2. To date, CAB2 has met virtually two times (May 2023 and October 2023). The early stages of CAB2 development have proven to be the most challenging. Snowball sampling, or the use of current participants to identify or connect with other potential participants [39], has been a useful technique to support recruitment. However, early turnover due to factors such as employment changes, outside commitments, or a lack of engagement, has presented barriers to both the retention of members and new recruitment through referrals from current participants. After our first two CAB2 meetings, our team experienced a change in the research team, as well as a delay in the scheduling of our third meeting. These issues contributed to the inability to hold a third meeting with CAB2, but efforts to reconstruct and continue both CABs were able to come together for the fourth and fifth meetings as one larger CAB.

### 3.2 Analysis Approach

Following meetings, audio recordings are transcribed and formatted for open coding. For data analysis, we utilise a constructivist grounded theory approach [40]. Constructivist grounded theory acknowledges the influence of researchers within the research process and promotes mutuality between researchers and participants [41]. Unlike traditional approaches to conducting focus groups, we as researchers consider ourselves to be active participants in the CABs [42]. Rather than asking unidirectional questions, we present findings and then ask and answer participants' questions. We expect that our presence and activities affect the data collected. A constructivist approach acknowledges this reality and encourages frequent reflexivity to identify the forms of this influence including researchers' own biases during collection and analysis. To do this, we use analytic memoing, or the process of recording questions or thoughts about how we may have influenced meetings throughout data analysis [19, 20].

Three researchers engage in coding all qualitative data from the CABs, individually assessing transcriptions through open‐coding and focused coding. Initial or open coding usually refers to the initial rounds of coding where all themes, large and small, are identified and recorded [43]. Focused coding is a re‐examination of the data and open codes for ‘parent codes’, or themes that encompass and relate multiple smaller codes [44]. During and after these rounds of coding, the coders meet to compare and draw connections between analyses. Finally, axial coding is used to examine the relationships between codes for potential mechanisms and intervention points [45]. The process is iterative, with data continually re‐examined throughout initial coding stages and after other meetings in a cumulative, participant‐driven, approach.

Unlike other methods such as traditional focus groups that view the data collected as the final product to be analysed, findings from CAB deliberations are then translated into actionable strategies for research design and other elements of the project. For example, in our project, CAB insights have been used to consider additional variables that should be included in conceptions of structural racism, or the measurement of specific barriers that patients face. For every research product that is created (i.e., manuscript, conference presentation, etc.), CAB insights are also integrated through contextualisation of findings and explicit connection to the implications of the study.

## 4 Results

### 4.1 Logistics

Meetings for both CAB1 and CAB2 occur in the following way: The first hour is spent reviewing preliminary findings and major themes from the previous meeting, doing brief re‐introductions of all participants and researchers, and presenting new quantitative findings (usually by the Principal Investigator of the project) of analyses that have occurred between meetings for comment and questions. Three out of the five rounds of meetings included these presentations; the initial meeting for each group was held to establish rapport between CAB members, and the most recent meeting focused explicitly on intervention ideas per CAB members' request. The second hour is spent discussing a list of semi‐structured questions guided by a continuation of lines of inquiry from previous meetings or that have emerged from quantitative analyses. During this time, participants are also invited to raise their own topics for discussion. It is essential to our approach to present all major research findings (regardless of the methodology or data used) from our project to the CAB before analyses are finalised or published. Community‐based research and CABs can take multiple forms depending on the role of researchers in CAB meetings (i.e., the researcher may act as an audience observer only or take an active role as a community contributor). In our case, the research team members take active roles through presenting and answering questions that CAB members may have. To ensure that this collaborative dynamic is possible and effective, researchers must remain open to learning from the CAB about how they perceive their role and how they would prefer to be integrated into the research paradigm.

Having patients and community members as research team members rather than just study participants allow for some flexibility methodologically. For example, as CAB members are not necessarily ‘being studied’ and have active influence over the research, in our experience CABs are not always subject to Institutional Research Board (IRB) approval. Instead, if one wants to pay members for their time, they can register as vendors (though the process likely differs by institution). However, this manner of registration for compensation is often tedious and quite complicated. Instead, one can choose to register them as study participants, seek IRB approval and then offer gift cards as compensation (which is the route we ultimately took).

### 4.2 CAB Integration in the Research Process

As previously described, traditional research analysis tends to be top‐down and may not involve and empower those most affected by the conditions being studied, leaving a glaring gap in research design. Indeed, there is a significant difference between conducting a focus group to gather data and inviting community members to inform integral parts of the research design as study partners. Whereas the former involves taking information provided by study participants (stated and/or inferred) to be used to inform research questions and objectives, the latter enables study participants to provide input into *how* this information can be used to shape research questions and objectives. In this project, the CAB serves as both a focus group and as study partners so that valuable qualitative data worthy of analysis is provided, while also helping to inform research questions, design and analysis.

The resultant CAB meetings have been used to inform the analysis and implications portion of research products from the project in multiple ways. While much of our quantitative data attempts to capture elements of racial disparities at an institutional level (i.e., politics, criminal justice, economics, segregation, health insurance), the CAB meetings help to delineate the mechanisms that connect this evidence of structural racism to observed racial disparities in ESKD. For example, we include a measure of education in our models. Discussions at the CAB meetings, as illustrated below, show exactly why education could be linked to disparities in treating ESKD:

For my father particularly… the highest level of education he has is the junior high school degree, and that's not to…say someone that has reading comprehension challenges or doesn't have anything higher than a junior high degree can't understand a doctor. But I think that his medical team should have understood that okay, these are the barriers that he experienced when he was younger in this system that led to where he is now. Is he understanding what we're telling him? And the answer is no. But they never took the time to understand that. I would ask my dad what his doctors were saying, he'd be like ‘I don't know. I just know I'm going to another surgery, or I'm going to the doctor's appointment’.

(Participant 10, Baltimore, May 2023)

These insights can also be directly linked to many of the CAB discussions regarding potential interventions. Consistently, the CAB suggestions have included a navigator system, or an external patient advocate that helps them navigate the complex world of kidney treatment. CAB members have provided countless examples of why these resources would be especially helpful to address structural racism throughout the ESKD process. Specifically, they have described the benefits that a navigator would have on issues particularly affected by structural racism including health literacy, access to information, and treatment decisions:

I've heard in many instances before the new CMS interventions, the centers would not necessarily get a lot of information around transplants. So, [patients] would really believe that they were consigned to being on dialysis for whatever length of time without the option to be able to actually have a transplant… The difficulty in really securing or getting all the information necessary to become a viable candidate becomes very difficult for that person to navigate without actually having navigators. I think in many of these communities, not having navigators that really understand specifically our challenges as a nonwhite community and assisting in that way creates a barrier all onto itself.

(Participant 8, Baltimore, May 2023)

This specific suggestion was applied to the implications of a paper focused on treatment sequences across the course of ESKD, which demonstrated significant racial/ethnic disparities in kidney replacement therapies by age, especially around deceased‐donor kidney transplants and home dialysis [46]. While prior research demonstrates the usefulness of patient navigator programs in chronic kidney disease (CKD) management [47, 48], CAB members' repeated mentioning of a navigator system to uniquely ease disparities has led to our team considering whether our quantitative data and analyses could support the use of patient navigators, as well as other interventions, for future efforts. We hope to identify when and for whom the recommended interventions will be most useful and practical in application.

### 4.3 Conceptions and Misconceptions

Other findings from the CABs help the research team to rethink concepts that have historically been used throughout disease management. For example, many patients who are on dialysis struggle to fully complete their weekly regimen [49]. Commonly referred to as ‘patient noncompliance’ throughout the medical community and literature, perceptions of ‘patient noncompliance’ can also be complicated by demographics of the patient [50], with patients of colour being frequently viewed as noncompliant [51, 52]. Deeply ingrained beliefs about noncompliance have globally shaped public health policies in racist ways [53]. Our work with the CABs demonstrates the importance of considering the structural elements that could be driving the difficulty adhering to treatments:

I'm legally blind. I have two vehicles outside. My daughter drives one. My wife drives the other. They all have active lives, so when I have doctors' appointments, I have to call for transportation to come pick me up. It's $10 one way, $10 back, plus the copay. I'm looking at about $70 per visit. If I have to go to the doctor's office three times a week, we're looking at over $200 in fees, not to mention any medicines that I might have to get.

(Participant 5, Pittsburgh, October 2023)

This lived experience provides needed contextualisation when reporting and analysing the specific impacts of structural racism, rather than chalking them up to previously accepted explanations. If the reasons for noncompliance can be identified, then strategies to alleviate them can be developed. Involving the community in these potential interventions is also a consistent suggestion of the CAB; they have expressed an interest in grass‐roots strategies that allow affected communities to contribute to easing disparities.

Additionally, an important point of working with CABs is that translating research findings in a digestible format is an important exercise to ensure that those findings can later be successfully communicated to the community broadly. Public health findings that indicate intervention points or delineate barriers provide important contributions; but only if they can be understood among those whom they impact. Our experiences with the CABs have demonstrated that the impacted community is more than capable of understanding the research if the information is communicated in an accessible way. Further, it is crucial that CABs be assembled and conducted in a way that is inclusive and not performative. CABs should not be considered a ‘box to check’ for researchers, which was reflected in our own realisation that community input was needed earlier and more frequently. Personal reflection and adherence to truly inclusive practices are necessary to fully realise the integration of these valuable lived experiences.

### 4.4 Challenges and Adaptations

Our initial approach included holding two separate CAB meetings, twice a year. However, we found that during the 6 months between meetings, research questions arose that would benefit from CAB input. To engage the CABs in real‐time, we intended to pilot a joint CAB where participants who are available and interested can assemble to provide real‐time feedback more frequently throughout the research process [3]. All members of the CABs expressed that they were interested in meeting more frequently. Before grant cancellation, we were going to shift to quarterly meetings instead of bi‐annual meetings. Should funding be re‐established, we intend to utilise quarterly meetings to increase the availability and integration of feedback. This approach comes with its own logistical and sampling challenges, as we acknowledge patients undergoing ESKD treatment already have significant time limitations, as do their caregivers and medical providers. However, more collaboration between CABs and greater integration of CAB members throughout the process produces research that places more emphasis on lived experiences. Such adjustments are often necessary in community engaged research [29].

We believe that increased engagement would help alleviate another challenge of ours, recruitment and retention. The Baltimore CAB was already established before our study's initiation, so it had a solid foundation on which to recruit additional individuals who were specifically affected by ESKD. This CAB has a myriad of connections between the members, including personal relationships that we have found to be both a strength for retention but also an uplifting experience when individual members get positive news about their own health journeys and feel comfortable sharing. The Pittsburgh CAB began with a few individuals who had participated in a CAB for a prior study, who were able to recommend others that are impacted by ESKD and may want to participate. However, the connections between members (and between members and the research team) were not as robust, leaving it more vulnerable to retention issues. To adapt to this, we have begun including interested Pittsburgh CAB members into Baltimore CAB meetings for a combined approach. As we have moved forward with the united approach, we have found significant benefits, especially as the number of members remained manageable.

A further challenge we have faced relates to incorporating presentations of the findings from quantitative analyses into the meetings for feedback without monopolising too much of the meeting. We have found it difficult to balance providing adequate context and background on the analyses to receive meaningful feedback from the CAB participants with leaving time to explore other topics with the group. Having multiple lines of inquiry competing for time, such as presenting findings and probing further into discussions raised in previous meetings, has limited data collection on both fronts. Additionally, it can be difficult to schedule meetings frequently enough while allowing for sufficient time for qualitative analysis. Therefore, analyses were occasionally presented at later stages for contextualisation, as well as policy and clinical implications. The intended shift in our approach to hold more frequent meetings was aimed at alleviating this problem and we recommend it for others who are integrating CABs into their projects.

Lastly, the largest barrier we now face is our NIH grant's termination due to the unsupported topics covered by this study project. The time of our CAB members is valuable, and we wish to continue fully compensating them for their contributions. Thankfully, the principal investigator's institution provided funds to cover CAB member honoraria for two additional meetings. As we expect the project to continue for more than a year, however, discussions on how to move forward in a way that honours the work already done, commitments made, and the importance of community involvement are ongoing.

## 5 Discussion and Conclusions

CABs center research on the lived experiences of patients and community members rather than the assumptions and predetermined expectations of researchers and funders. Quantitative analyses can help us illuminate the ‘what’ within research inquiries, while incorporating qualitative lived experiences of those impacted by health conditions helps shed light on the mechanisms contributing to the development and progression of conditions beyond what is expressed in medical records (the broader ‘how’ and the ‘why’). For our purposes, the lived experiences of those who have first‐hand knowledge of ESKD inform all phases of our research. We also recognise that ESKD affects the lives not only of a person with kidney disease, but also their friends and family, caregivers, living kidney donors and the broader communities. It is through the inclusion of individuals who represent these roles that we hope to bring humanistic and person‐focused perspectives to our research.

Our CAB discussions have led to important insights on the mechanisms that connect observed structural racism at county and state levels to the lived experience of individuals of colour with ESKD. Operating under a definition of structural racism as resulting in ‘observable…racial inequalities in life chances’ [18, 19], we seek to inform both the measurement of the problem and contextualise the mechanisms being observed. It is well known that socioeconomic disenfranchisement is an important element of structural racism [19], and that despite the contributions of Medicare coverage for ESKD, socioeconomic status is closely tied to ESKD outcomes [54]. The CABs reflections on specific barriers such as transportation to dialysis appointments and access to transplants (which can be extremely costly for the donor and the recipient due to resources needed for time off work, insurance and recovery) help shed light on the specific links between socioeconomic status, structural inequities and ESKD.

It has become apparent to our research team that affected community members are hungry for a solution to racial/ethnic disparities in ESKD, not just better evidence about the problem. Therefore, we have prioritised explicitly drawing links between analyses demonstrating the scope of the problem of structural racism to suggested interventions. Specifically, we are focusing on barriers to accessing transplants, a prominent theme arising throughout CAB discussions about potential interventions. Frequently, the CAB discussed perceptions of interpersonal discrimination that may be conditioning access to transplants, especially around recipient ordering for an available kidney, and initial referral to transplant centres. We are applying advanced quantitative techniques such as sequence analysis and matching estimator analyses to pinpointing which transitions in the process exhibit the highest levels of racial/ethnic disparities, which will hopefully provide necessary information for designing interventions. Additionally, CAB discussions around a dearth of pre‐ESKD nephrological care has reshaped our approach to studying the role of structural racism in racial/ethnic disparities in the CKD to ESKD transition. Such insights have been used to develop new intervention‐focused research questions on community‐based educational campaigns about the signs and symptoms of CKD and the importance of early care.

Involving patients and community members in studies of the sociological elements of disease is crucial, as lived experiences provide fundamental information that can be missed by medical records and arms‐length analyses [55]. However, significant barriers to the successful implementation of community advisory boards exist. Active participation in these boards places additional time and resource loads on individuals who are already undergoing challenging circumstances. Therefore, throughout the process we have sought to minimise the time demands for CAB participants and maximise the value of each meeting. A unique challenge for this approach includes guarding against only presenting findings near the completion of a line of inquiry for input in latter stages, instead incorporating CAB members earlier on in the process to guide future research directions as well. While funding limitations may further complicate the use of CABs for public health research, all attempts should be made to continue the goal of community‐involved research, especially for marginalised populations.

## Author Contributions

**Andrea Corradi:** conceptualisation (lead), writing – original draft preparation (lead), methodology (Equal), investigation. **Grecia B. V. Meléndez:** writing – original draft preparation, investigation. **Tanjala S. Purnell:** supervision (equal), investigation, writing – reviewing and editing. **Selena E. Ortiz:** Methodology (equal), writing – reviewing and editing. **Catherine R. Butler:** writing – reviewing and editing. **Jonathan Daw:** supervision (equal), writing – reviewing and editing.

## Ethics Statement

The Institutional Review Board at Johns Hopkins School of Medicine approved the project activities as exempt from review (IRB00187277).

## Conflicts of Interest

The authors declare no conflicts of interest.

## Data Availability

The data that support the findings of this study are available on request from the corresponding author. The data are not publicly available due to privacy or ethical restrictions.

## Endnotes

1 Funding was revoked in May 2025.
